# Application of ^82^Sr/^82^Rb generator in neurooncology

**DOI:** 10.1002/brb3.1212

**Published:** 2019-02-06

**Authors:** Nikolay A. Kostenikov, Boris L. Zhuikov, Valeriy M. Chudakov, Yuriy R. Iliuschenko, Sergey V. Shatik, Vadim V. Zaitsev, Dmitriy S. Sysoev, Andrey A. Stanzhevskiy

**Affiliations:** ^1^ Russian Research Center of Radiology and Surgical Technologies (RRCRST) of Ministry of Public Health Saint‐Petersburg Russia; ^2^ Institute for Nuclear Research of Russian Academy of Sciences (INR RAS) Moscow Russia

**Keywords:** ^82^Sr/^82^Rb generator, brain tumors, PET, ^82^Rb‐chloride, radiopharmaceutical

## Abstract

**Introduction:**

The applicability of “Rubidium Chloride, ^82^Rb from Generator” radiopharmaceutical for brain tumors (BT) diagnostics is demonstrated on the basis of the application experience of the radiopharmaceutical in neurooncology.

**Experimental:**

A total of 21 patients with various brain tumors and nonneoplastic abnormal brain masses were investigated.

**Results and Discussions:**

The results of the imaging and differential diagnostics of malignant and benign tumors, nonneoplastic abnormal brain masses and lesions revealed the prevalence of high uptake of the radiopharmaceutical in the malignant tumors in comparison with benign glioma and arteriovenous malformations in which ^82^Rb‐chloride accumulates in the vascular phase but does not linger further. The ultra‐short half‐life of radionuclide ^82^Rb (76 s) along with a low absorbed radiation dose with ^82^Rb‐chloride by intravenous administration create a new possibility of successive use of two or more radiopharmaceuticals for the examination of the same patient. For instance, PET examination with ^18^F‐FDG, ^11^C‐methionine, ^11^C‐choline, or any other radiopharmaceutical can be carried out in just 7–15 min. after ^82^Rb‐chloride injection.

**Conclusion:**

Research demonstrated an effectiveness of ^82^Rb‐chloride application as a diagnostic agent in neurooncology. A method of dosing and administration of the generator‐produced radiopharmaceutical has been worked out. It is possible to do up to 600 PET sessions using one Russian ^82^Rb generator GR‐01. The generator is proved to be reliable and easy to use. The interest in ^82^Rb‐chloride as a tumor‐seeking radiopharmaceutical rose due to the active application of the modern devices PET/CT in the routine clinical practice.

## 1 INTRODUCTION

The references of using ^82^Sr/^82^Rb generator (^82^Rb generator) for positron‐emission tomography (PET) dates back to the 70 s (Kulprathipanja, Hnatowich, & Ben, 1979; Yano & Roth, 1979). Generator for clinical use based on hydrated tin oxide were developed in the USA (Neidrickx, 1980), the UK (Waters, Horlock, & Kensett, 1983), Switzerland (Beyer, Rӧsch, & Ravn, 1990), Canada (Cackette, Ruth, & Vincent, 1993), Russia (Kodina, Kurenkov, Kurchatova, Malinin, & Yu, 1991; Litvinov et al., 1997), and Korea (Jeong et al., 1994). At present, the ^82^Rb‐generators are mainly produced in the USA by GE Healthcare and distributed by Bracco Diagnostics Inc. (CardioGen^®^) (Bracco Diagnostics^2000^‐2000, 2000) (approved by FDA in 1989), as well as recently in Canada by Jubilant DraxImage (Ruby‐Fill^®^) (Jubilant DraxImage, 2018) (approved by FDA in 2016). The Russian ^82^Rb generator GR‐01 which is produced and used at the clinic of the Russian Research Center of Radiology and Surgical Technologies of Ministry of Public Health (RRCRST, St. Petersburg) was developed in the Institute for Nuclear Research of Russian Academy of Sciences (INR, Moscow) with the participation of TRIUMF (Vancouver, Canada) (Chudakov et al., 2005, 2014; Chudakov, Zhuikov, & Ermolaev, 2015; Chudakov, Zhuikov, & Kokhanuk, 2015). A new approach of ^82^Rb clinical application for PET 002Ddiagnostics in cardiology and later in oncology was developed in RRCRST in 2005–2007 (Granov, Matveev, Zhuikov, Kostenikov, & Rizhkova, 2011; Granov, Tyutin, & Kostenikov, 2012; Granov, Tyutin, Tlostanova, Rizhkova, & Kostenikov, 1997). Preclinical and clinical trials of the ^82^Rb generator were carried out in the RRCRST in 2005–2011. The ^82^Rb generator was registered as a medical device by Federal Service for Surveillance in Healthcare of Russian Federation in 2014 (Registration certificate of medical production No RZN 2014, 2014).

The generator‐produced radiopharmaceutical has an important advantage: it does not require a complicated and expensive cyclotron–radiochemical complex.

Due to a short half‐life of ^82^Rb (76 s), the absorbed dose of radiation is low for a patient. It gives the opportunity to repeat testing many times as well as to use several radiopharmaceuticals successively within one day for the same patient.

Earlier the generator was used almost in cardiology only (Chatal et al., 2015). The first study of brain tumors perfusion of using PET with ^82^Rb‐chloride was carried out in 1980–90 s (Brooks et al., 1984; Roelcke et al., 1996; Yen et al., 1982). The attention was paid to ^82^Rb^+^ property to penetrate the blood‐brain barrier (BBB).

In particular, some authors studied ^82^Rb‐chloride diagnostic capabilities during the estimation of blood‐brain barrier permeability in patients with glial tumors and metastatic brain lesions (Brooks et al., 1984). The obtained results showed the capacity of ^82^Rb‐chloride to higher uptake in the areas with a damaged blood‐brain barrier. The diagnostic accuracy of the method was similar to the data of the perfusion X‐ray computed tomography (PCT) and magnetic resonance perfusion imaging (MRI).

Advantages and disadvantages of MRI and PET in perfusion study are not yet well investigated. At perfusion investigation with MRI, the extent of analyzed zone of interest is limited by the size of the field of view of the tomograph. This makes difficult to scan necessary volume of human brain in some cases.

Susceptibility artifacts, especially in zones of the frontal sinus and ethmoid labyrinth may appear (Jarnum et al., 2010) at MRI investigation. The results of measurement are very sensitive to technical parameters of the scanner. The proposed method of PET investigation with ^82^Rb‐chloride is free from these disadvantages.

An information obtained with ^82^Rb‐chloride PET investigations is especially effective in combination with contrast‐enhanced MRI using PET/MRI fusion technique. In this case, a final conclusion on the role and advantage of PET with ^82^Rb‐chloride in brain tumor studies may be drawn (Nensa, Beiderwellen, Heusch, & Wetter, 2014).

This approach is simple, reproducible, and provides obtaining exact information on tumor perfusion. Short period of ^82^Rb half‐life provides low radiation doses. The authors set protocols for the PET data acquisition and mathematical models to calculate a constant, which shows the rate of intracellular transportation of rubidium‐82 cations into the brain tumor cells (Brooks et al., 1984).

The interest in ^82^Rb‐chloride as a tumor‐seeking radiopharmaceutical rose 2010–2011 due to the active application of the new multimodal device PET‐CT in the routine clinical practice (Kale, Halkar, & Galt, 2007; Khandani, Sheikh, Beavers, & Ivanovic, 2010; Mirpour & Khandani, 2011). So far, there are few reports (Brooks et al., 1984; Kale et al., 2007; Khandani et al., 2010; Mirpour & Khandani, 2011; Roelcke et al., 1996; Yen et al., 1982) on the capability and diagnostic efficiency of ^82^Rb‐chloride in neurooncology. But in these researches, the authors mainly focus on comparing ^82^Rb‐chloride and ^11^C‐methionine without paying much attention to the significant aspect mentioned above.

The comparison of ^82^Rb‐chloride diagnostic capability with those of ^18^F‐FDG is considered highly important, since the two radiopharmaceuticals provide mutually complementary data concerning properties of the malignant tissue under examination. This gives the opportunity to estimate both tumor perfusion (^82^Rb‐chloride) and tumor metabolism (^18^F‐FDG).

The study of ^82^Rb accumulation/elimination in/out of the tumor based on the analysis of the activity/time curves above the tumor is also of significant interest for diagnostic purposes. Such an attempt was made in (Roelcke et al., 1995) using ^82^Rb‐chloride, ^11^C‐methionine, and ^18^FDG as well as (Valk et al., 1988) using ^82^Rb‐chloride and ^18^FDG. But detailed analysis of the obtained activity/time curves has not been carried out by the authors probably due to too long time of ^82^Rb‐chloride injection and short time of PET scanning.

The application of diagnostics with ^82^Rb‐chloride may be an important independent method in PET investigation of brain, or, in particular, a complementary method to ordinary approaches (Bell et al., 2015; Herholz, 2017) using compounds with ^18^F, ^11^C, and ^13^N, especially in combination with cardiological application of the same generator.

The goal of the current research is obtaining more data with ^82^Rb PET in combination with FDG PET and different diagnostic methods (CT, MRI and angiography). The present study is a pilot study, and more data need to be accumulated.

## 2 EXPERIMENTAL

The procedures followed were performed in accordance with the Helsinki Declaration of 1975, as revised in 2008. The Study Protocol, Investigator's Brochure, and Summary of Product Characteristics were approved by the institutional Review Ethics Board of RSCRCT as well as by Federal Service for Surveillance in Healthcare (Roszdravnadzor, Russian Federation). All patients (human subjects) provided written informed consent prior to participation in this study.

**Figure 1:**
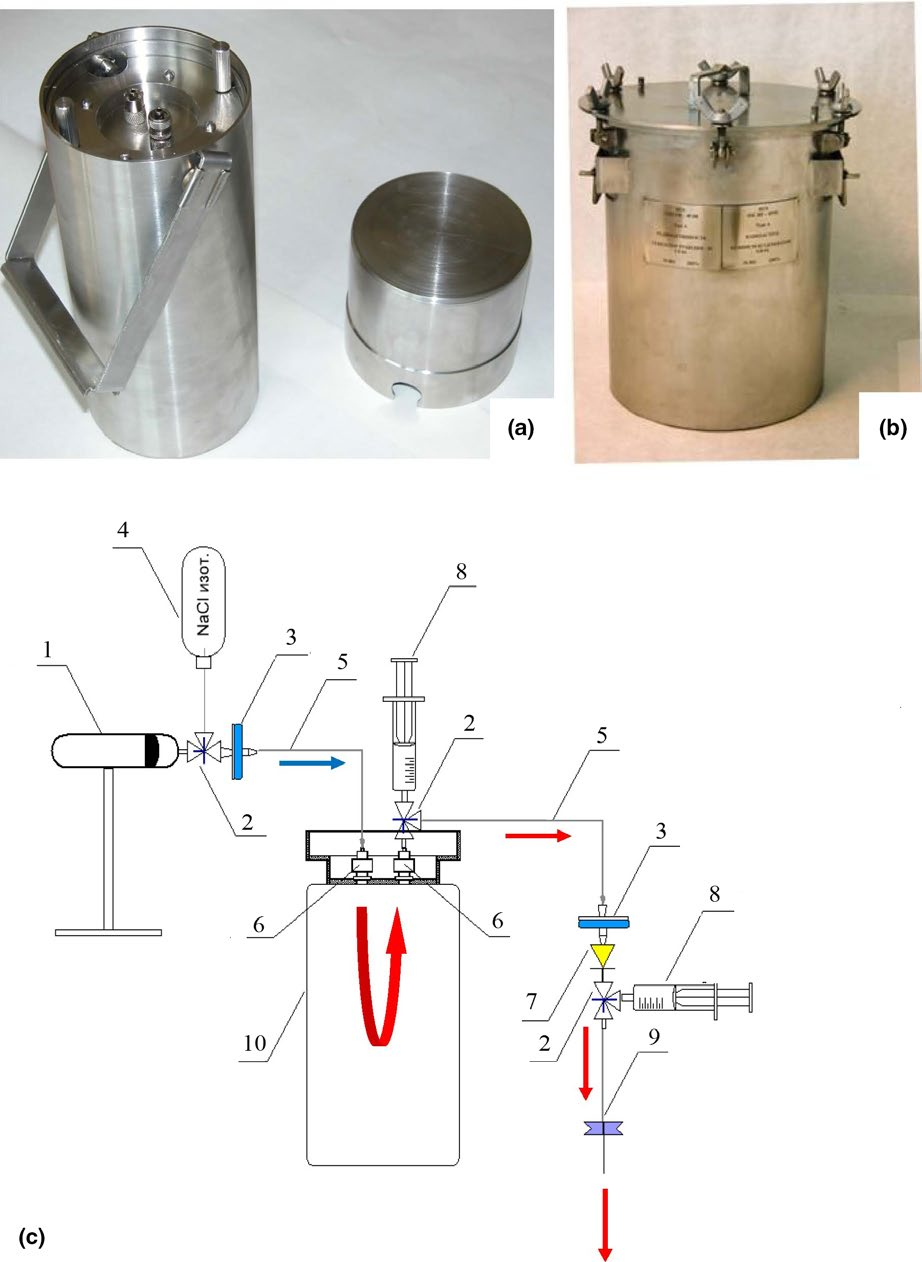
(a) ^82^Sr/^82^Rb generator GR‐01 (left). (b) Package Design. (c) ^82^Rb‐chloride radiopharmaceutical elution system scheme: Programmable syringe pump—1; 3‐way stopcock—2; sterilizing filter—3; saline supply—4; plastic extender (tubing with Luer edges)—5; adapter (from Swagelok to Luer)—6; one‐way valve—7; syringe with saline for air removal from the system—8; peripheral venous catheter—9; ^82^Sr/^82^Rb generator—10

^82^Rb‐generator GR‐01 with package design and ^82^Rb elution system scheme is shown in Figure 1. The generator described in details in (Chudakov et al., 2014) consists of a generator column encased in a shielding container made of tungsten and steel (Figure 1a). The column contains a parent radionuclide ^82^Sr (T_1/2_ = 25.55 d) adsorbed on the ion‐exchange material based on hydrated tin oxide. A daughter radionuclide ^82^Rb (T_1/2_ = 76 s) generated as decay product of ^82^Sr is eluted from the generator in the isotonic solution (0.9% NaCl). Up to 6 GBq (160 mCi) of ^82^Sr may be loaded into the generator GR‐01. For transportation of ^82^Sr/^82^Rb‐generator, a certified package design is used (Figure 1b). The scheme of elution system is shown in Figure 1c (the blue arrow indicates the saline flow direction, and the red arrows indicate the flow direction of eluate with ^82^Rb‐chloride). A minimum time to perform sequential imaging in studies of patients is 15 min.

There are three possible elution types of radiopharmaceutical administration: constant‐flow elution, constant‐activity elution, and constant‐time elution. For the administration of the generator‐produced radiopharmaceutical ^82^Rb‐chloride, we use the constant‐time elution as the most preferable one. As a rule, a stream infusion of the radiopharmaceutical lasts 14 s (the period from 11 to 16 s is acceptable). The volume of eluate per infusion ranges from 5 to 25 ml, and the infusion rate ranges from 3 to 90 ml/min (the usual range is 40–80 ml/min). Provided service conditions, the entire period of the generator operation ranks over all known analogs and certified for 60 days. A possible eluate volume before the critical breakthrough of ^82^Sr (0.01 kBq/MBq ^82^Rb) or ^85^Sr (0.1 kBq/MBq ^82^Rb) is up to 30 L in GR‐01 generator.

2D positron‐emission tomography “Ecat‐Exact‐HR+” (Siemens AG Germany) was used for the investigations. According to the regular practice, in cardiological tests for diagnostic purpose the administered dose of ^82^Rb‐chloride ranges from 2,000 to 2,500 MBq. In this research, the diagnostic dose of ^82^Rb‐chloride radiopharmaceutical in neurooncological patients was defined as about 800 MBq per 1 m^2 ^of patient's body and makes up 820–1,500 MBq per single injection in order to get enough good images with 2D‐scanner. A coefficient for calculating an effective dose is 1.26 μSv/MBq using the tissue‐weighting factor of the International Commission on Radiological Protection (International Commission on Radiological Protection (ICRP), 2008). So, the total effective dose was 1.0–1.9 mSv per single injection in compare, for example, with about 7–9 mSv from injected ^18^F‐FDG activity 370–450 MBq (Marti‐Climent et al., 2017; Quin, Dauer, Pandit‐Taskar, Scholder, & Dauer, 2016).

This fact stimulates using ^82^Rb‐chloride after application of the same generator in cardiological investigations when ^82^Sr‐activity in the generator drops down and is too low for obtaining good images in cardiology.

Special preparation of patients was not required. Before the examination, 10‐min transmission scanning with ^68^Ge calibration source was performed for attenuation correction. We performed emission scanning in “2D‐mode” with 128 × 128 matrix and zoom image by 1.5. The scanning was carried out in the dynamic mode: 6 frames at 10 s each, 4 frames at 30 s each, 4 frames at 60 s each, and 2 frames at 120 s each (11 min in total). Total study duration takes approximately 30 min. per one patient (the inclusion period was from 4 February to 18 December 2015). The image reconstruction was done with “Backprojection”‐filter and zoom image by 2.5. The region of interest in the brain reconstructed image was detected according to the data on brain lesion location obtained by structured methods (MRT). Frames starting from the end of bolus elution (1 min) up to the end of the uptake phase were summed (from 7th to 20th frame) in tissue phase. The uptake index (UI) and vascularization degree (VD) were estimated, and activity/time curves for tumors and normal brain cortex areas were plotted. UI is a ratio between maximal activity value accumulated in zone of interest of the tumor and maximal activity value in the zone of interest of the intact brain tissue. VD is a ratio between maximal activity value accumulated in zone of interest of the tumor and maximal activity value in the normal cortex of the vascular phase.

A total of 21 patients at the age of 18–76 with different brain tumors were examined. All the patients underwent PET examination with ^82^Rb‐chloride along with MRT with contrast agent, and some patients underwent PET examination with ^18^F‐FDG, CT along with brain X‐ray angiography.

## 3 RESULTS AND DISCUSSIONS

In 14 of total amount, 21 patients (67%) brain tumors were diagnosed. In 10 of 14 patients, malignant tumors (8 glioblastoma and 2 meningioma) were diagnosed. In other 4 of 14 patients, the benign brain tumors (3 astrocytoma and 1 meningioma) were diagnosed. In 3 of 21 patients, postsurgical cysts and in 4 patients arteriovenous malformations were diagnosed.

**Figure 2:**
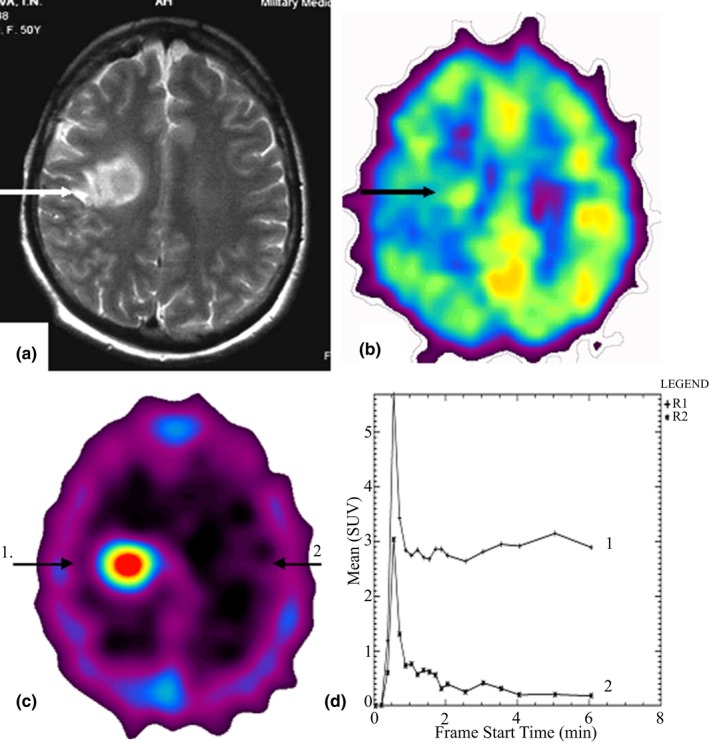
(a‐d), Female patient g. Diagnosis: glioblastoma multiforme recurrence growth of the right parietal lobe. (a) At MRI (T2‐weighted image), the tumor is well visible, and perifocal edema is defined. (b) ^18^F‐FDG PET image demonstrates heterogeneous unsharp mass with the high glycolytic rate glycolysis (UI = 1.0). The lesion is partially visible due to its location in the white matter as well as to edema and ischemization of the adjacent brain cortex. (c) ^82^Rb‐chloride PET image demonstrates a sharp homogeneous hypervascular focus with high radiopharmaceutical uptake in the tissue phase (UI = 17) (position 1). Contralateral brain cortex is shown in position 2. (d) Activity/Time curve of the lesion (1) shows that the tumor has the high vascularization degree (VD = 2.1). Vascular permeability is impaired. Within the tissue phase, high radiopharmaceutical uptake in tumor exceeds the radiopharmaceutical uptake in the contralateral brain cortex (2) in 17 times. A typical for malignant tumors slow monotonic radiopharmaceutical uptake in the tumor within the tissue phase is observed

**Figure 3:**
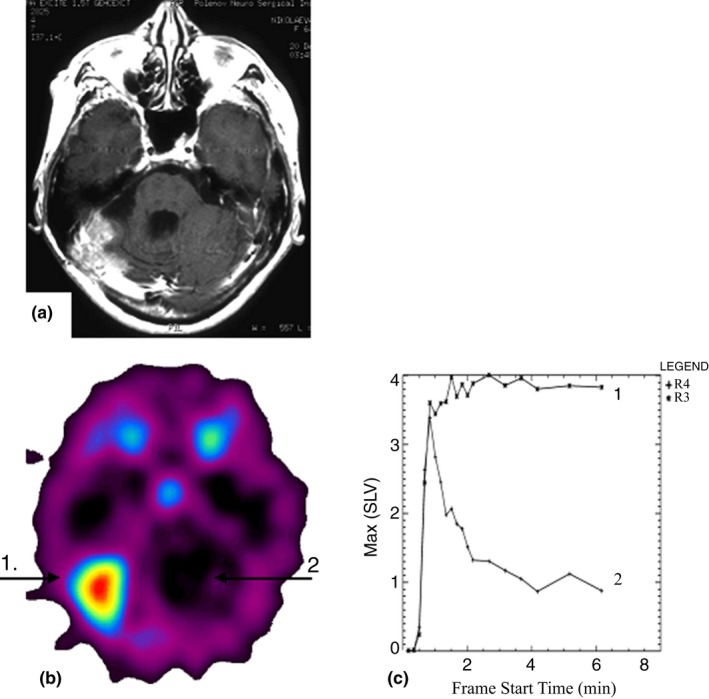
(a‐c), Female patient n. Diagnosis: anaplastic meningioma recurrence growth of the posterior cranial fossa on the right. (a) At the CT image, the lesion is not well visualized, and the boundaries are unsharp. (b) ^82^Rb‐chloride PET image demonstrates a homogeneous mass of hyperfixation with high radiopharmaceutical uptake within the tissue phase (UI = 9.7) (position 1). Contralateral brain cortex is shown in position 2. (c) Activity/Time curve of the tumor (1) shows that the lesion has moderate vascularization. Vascular permeability is impaired. A typical for malignant lesions low monotonic radiopharmaceutical uptake in the lesion within the tissue phase is observed. Relatively low vascularization for a meningioma could be explained by preceded treatment. The activity/time curve (2) over contralateral cortex is shown for comparison

In all 10 cases, malignant tumors (glioblastoma and meningioma) were exactly visualized at PET examination with ^82^Rb‐chloride. In all the cases, abnormal focuses of radiopharmaceutical high uptake were detected in the image of a tumor node (Figures 2, 3). Activity/time curves plotted over malignant tumors images (Figure 2) show for high vascularization of the malignant tumors along with pathological permeability of cell membranes of the tumor blood vessels as compared to intact brain cortex. It is noteworthy that as a rule, monotonic increase in radiopharmaceutical's uptake in malignant glioma and meningioma was detected during the whole scan session (11 min) (Figures 2, 3).

Earlier in (Granov et al., 2011, 2012, 1997), we demonstrated that optimal time for PET scanning with ^82^Rb‐chloride is 11 min. Along with this, in order to obtain correct activity/time curves the radiopharmaceutical should be administrated during 11–16 s which is faster than in (Roelcke et al., 1995) (30–60 s when scanning time is 6 min).

**Figure 4:**
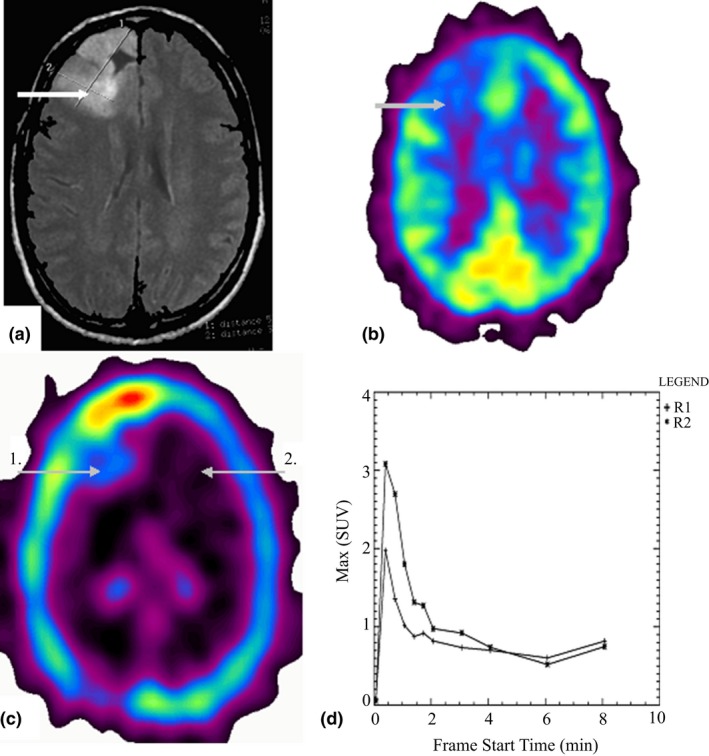
(a‐d) Male patient i. Diagnosis: astrocytoma of low malignant potential (Gr II). (a) MRI T1 WI image demonstrates a space‐occupying lesions of the anterior convexital parts of the right frontal lobe. (b) ^18^F‐FDG PET image demonstrates that the lesion is partially visualized due to its white matter location as well as to the perifocal edema, and the level of the glucose metabolism is low (UI = 0.6). (c) ^82^Rb‐chloride PET image demonstrates the isovascular focus with low radiopharmaceutical uptake (UI = 1.2) in the projection of the lesion continued growth (position 1). Contralateral brain cortex is shown in position 2. (d) Activity/Time curve obtained in ^82^Rb‐chloride PET shows that the lesion (1) has moderate vascularization degree (VD = 1.6). The fact that radiopharmaceutical uptake is weak and low and radiopharmaceutical does not linger in the tissue phase indirectly points at the low malignant potential of the lesion. The activity/time curve (2) over contralateral cortex is shown for comparison

Radiopharmaceutical uptake in the tumor node appeared to be low in 3 patients with astrocytoma of a low malignant potential (Figure 4). Tumor imaging in such cases became possible entirely due to the far lower uptake of ^82^Rb‐chloride in intact brain cortex, and due to this fact, tumors were partially seen (Figure 4). As a rule, within the tissue phase monotonic radiopharmaceutical elimination was observed or radiopharmaceutical content remained at low level. At the same time, high radiopharmaceutical uptake in the tumor was detected in both vascular and tissue phases in the patient with benign meningioma, and this fact may be explained by high vascularization of meningioma.

Radiopharmaceutical uptake in postsurgical cysts after tumor eradication was close to its background absorption. Cysts were not visualized on the background of radiopharmaceutical low uptake in the brain cortex.

**Figure 5:**
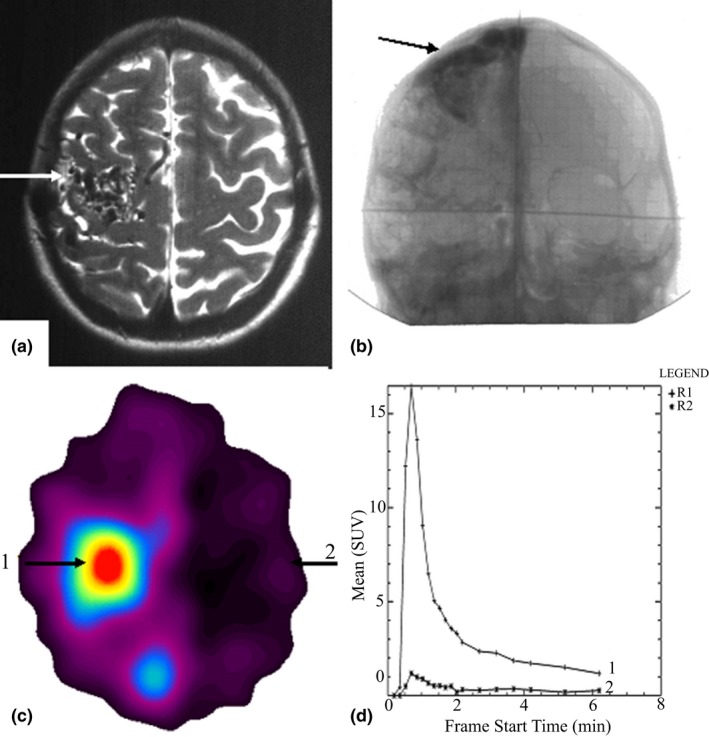
(a‐d) Female patient Z. Diagnosis: arteriovenous malformation of the right parietal lobe. (a) MRI (T2‐weighted image) showing that AVM is well visible. (b) The angiography (in sagittal slice) shows that AVM is well visible, a developed vast vasculature of the aneurysm is observed. (c) Under the ^82^Rb‐chloride PET examination, we observed a heterogeneous hypervascular focus (position 1), that was swell‐visualized within the tissue phase, but the radiopharmaceutical uptake within the vascular phase was low (UI = 1.1–1.2). Contralateral brain cortex is shown in position 2. (d) Activity/Time curve of AVM shows that the malformation has high vascularization degree (VD = 14), but radiopharmaceutical rapid biexponential clearance took place in the tissue phase. The activity/time curve (2) over contralateral cortex is shown for comparison

In patients with arteriovenous malformation (AVM), high ^82^Rb‐chloride uptake was detected only in the vascular phase and it was low in the tissue phase (Figure 5).

An uptake index UI (ratio ^82^Rb‐uptake in tumor/^82^Rb‐uptake in normal brain tissue) was estimated in order to carry out the quantification of obtained data. Obtained results of the radiopharmaceutical uptake level in the examined masses are shown in Table 1.

**Table 1:** Radiopharmaceutical ^82^Rb‐chloride uptake level in the brain lesions (21 patients in total)

Morphological diagnosis	Number of patients	Uptake index (UI) for brain lesions, tissue phase (observed range and average)	Vascularization degree (VD) for brain lesions, of vascular phase (observed range and average)
Glioblastoma multiform (GrIV)	8	17.9–25.1 21.6 ± 5.3	1.8–2.5 2.07 ± 0.26
Benign astrocytoma	3	1.1–1.4 1.25 ± 0.15	1.2–1.7 1.46 ± 0.22
Malignant meningioma (GrIV)	2	9.7–11.2 10.5 ± 1.1	2.1–2.2 2.15 ± 0.05
Benign meningioma	1	10.5	2.5
Arteriovenous malformation (AVM)	4	1.2–2.2 1.8 ± 0.45	11.2–17.8 14.5 ± 2.4
Postsurgical cyst	3	0.4–1.5 0.7 ± 0.6	0.4–1.0 0.47 ± 0.17

Note

UI is a ratio between maximal activity value accumulated in zone of interest of the tumor and maximal activity value in the zone of interest of the intact brain tissue. VD is a ratio between activity value accumulated in region of interest in the tumor and activity value in the intact brain tissue of vascular phase.

The data represented in Table 1 show that the high ^82^Rb‐chloride uptake (in UI units) was observed in malignant tumors. On average, UI found in malignant tumors was 19.2 ± 5.8, while in benign glioma and postsurgical cysts the radiopharmaceutical uptake was significantly lower (Student's coefficient *p* < 0.05). The high radiopharmaceutical uptake in malignant glioma made it possible to rule them out from astrocytoma of low malignant potential (*p* < 0.05). A distinct direct correlation was revealed (Spearman Rank *r* = 0.79) between the perfusion level and malignancy grade of the glial tumor under PET investigation with ^82^Rb‐chloride. The high uptake of ^82^Rb‐chloride in both malignant and benign meningiomas was observed due to the fact of their high vascularization.

Therefore, the high radiopharmaceutical uptake in malignant glioma and low radiopharmaceutical uptake in benign glioma are associated with the tumor vascularization grade. The fact makes it possible to use PET examination with ^82^Rb‐chloride for differential diagnostics between malignant and benign glioma. It also gives the opportunity not only to image malignant lesions but also to rate glial lesions according to their malignant grade.

^82^Rb‐chloride is a nonspecific radiopharmaceutical, and for this reason, the level of its uptake by the tumor primarily depends on the tumor vascularization. This fact was demonstrated in comparison with malignant and benign gliomas: it was possible to differentiate between benign and malignant gliomas. The high radiopharmaceutical uptake in benign meningioma made it impossible to differentiate between benign and malignant meningiomas. However, this problem can be easily solved by successive using ^18^F‐FDG which does not accumulate in benign tumors. Also, the differential diagnostics of meningioma is generally made during MRI examination.

An important problem is what mechanisms could contribute to ^82^Rb accumulation in brain tumors. The level of ^82^Rb‐uptake in tumor directly depends on its vascularization rate, integrity of blood‐brain barrier (BBB), and sodium–potassium pump efficiency. Vascularization, BBB disruption, and efficiency of sodium–potassium pump are considerably higher in malignant tumors in comparison with benign tumors. Thus, Rb (as an analogue of potassium) penetrates BBB from extracellular fluid and is more easily retained in cells in comparison with benign glioma, especially if the pump functioning is incorrect. This may be a reason for ^82^Rb hyperfixation and incorrect functioning of the pump. A detailed future study with more quantitation of images will provide more efficient application of ^82^Rb‐chloride pharmaceutical in neurooncology.

## 4 CONCLUSION

1. Application of radiopharmaceutical ^82^Rb‐chloride from ^82^Sr/^82^Rb generator demonstrates a possibility of diagnostics different tumor and nontumor lesions, such as AVM, in human brain with low radiation effective doses (1.0–1.9 mSv).
2. Uptake index and activity/time curve analysis in constant‐elution time mode with short‐lived ^82^Rb (T_1/2_ = 76 s) provide evaluation of vascularization in tumors and malignancy of gliomas.
3. A low diagnostic dose acceptable in neurooncological studies (up to 820 MBq with 2D‐scanner) makes it possible to use ^82^Sr/^82^Rb generator during a long period (60 days certified for generator GR‐01), in particular, after cardiological studies with the same generator.

## CONFLICT OF INTERESTS

The authors have no conflict of interests to declare.
